# Concordance Between Genomic Alterations Detected by Tumor and Germline Sequencing: Results from a Tertiary Care Academic Center Molecular Tumor Board

**DOI:** 10.1093/oncolo/oyac164

**Published:** 2023-01-18

**Authors:** Michelle F Green, Catherine H Watson, Sarah Tait, Jie He, Dean C Pavlick, Garrett Frampton, Jinny Riedel, Jennifer K Plichta, Andrew J Armstrong, Rebecca A Previs, Noah Kauff, John H Strickler, Michael B Datto, Andrew Berchuck, Carolyn S Menendez

**Affiliations:** Department of Pathology, Duke University Medical Center, Durham, NC, USA; Department of Obstetrics and Gynecology, Duke University, Durham, NC, USA; Duke University, School of Medicine, Durham, NC, USA; Foundation Medicine, Inc., Cambridge, MA, USA; Foundation Medicine, Inc., Cambridge, MA, USA; Foundation Medicine, Inc., Cambridge, MA, USA; Duke Cancer Institute, Duke University Medical Center, Durham, NC, USA; Department of Surgery, Duke University Medical Center, Durham, NC, USA; Duke Cancer Institute Center for Prostate and Urologic Cancers, Duke University, Durham NCUSA; Department of Obstetrics and Gynecology, Duke University, Durham, NC, USA; Duke Cancer Institute, Duke University Medical Center, Durham, NC, USA; Duke Cancer Institute, Duke University Medical Center, Durham, NC, USA; Division of Medical Oncology, Department of Medicine, Duke University Medical Center, Durham, NC, USA; Department of Pathology, Duke University Medical Center, Durham, NC, USA; Department of Obstetrics and Gynecology, Duke University, Durham, NC, USA; Department of Surgery, Duke University Medical Center, Durham, NC, USA

## Abstract

**Objective:**

The majority of tumor sequencing currently performed on cancer patients does not include a matched normal control, and in cases where germline testing is performed, it is usually run independently of tumor testing. The rates of concordance between variants identified via germline and tumor testing in this context are poorly understood. We compared tumor and germline sequencing results in patients with breast, ovarian, pancreatic, and prostate cancer who were found to harbor alterations in genes associated with homologous recombination deficiency (HRD) and increased hereditary cancer risk. We then evaluated the potential for a computational somatic-germline-zygosity (SGZ) modeling algorithm to predict germline status based on tumor-only comprehensive genomic profiling (CGP) results.

**Methods:**

A retrospective chart review was performed using an academic cancer center’s databases of somatic and germline sequencing tests, and concordance between tumor and germline results was assessed. SGZ modeling from tumor-only CGP was compared to germline results to assess this method’s accuracy in determining germline mutation status.

**Results:**

A total of 115 patients with 146 total alterations were identified. Concordance rates between somatic and germline alterations ranged from 0% to 85.7% depending on the gene and variant classification. After correcting for differences in variant classification and filtering practices, SGZ modeling was found to have 97.2% sensitivity and 90.3% specificity for the prediction of somatic versus germline origin.

**Conclusions:**

Mutations in HRD genes identified by tumor-only sequencing are frequently germline. Providers should be aware that technical differences related to assay design, variant filtering, and variant classification can contribute to discordance between tumor-only and germline sequencing test results. In addition, SGZ modeling had high predictive power to distinguish between mutations of somatic and germline origin without the need for a matched normal control, and could potentially be considered to inform clinical decision-making.

## Teaser

This study sought to describe concordance between germline and tumor testing among patients at the investigators' institution and queried whether results from one test type could be used to inform the other. The authors also investigated the potential of a computational modeling algorithm to predict germline status based on tumor-only comprehensive genomic profiling results alone.

**Implications for Practice**

The majority of tumor sequencing currently performed on cancer patients does not include a matched normal control, and in cases where germline testing is performed, it is usually run independently of tumor testing. The rates of concordance between variants identified via germline and tumor testing in this context are poorly understood. This study found that mutations in cancer predisposing genes identified by tumor-only sequencing are frequently germline, but providers should be aware that technical differences related to assay design, variant filtering, and variant classification can contribute to discordance between tumor-only and germline sequencing test results. In addition, computational somatic-germline-zygosity (SGZ) modeling had high predictive power to distinguish between mutations of somatic and germline origin without the need for a matched normal control, and could potentially be considered to inform clinical decision-making.

## Introduction

Homologous recombination is a complex process involving multiple genes that function to repair DNA double-strand breaks to maintain genome stability.^1^ Identification of a pathogenic germline mutation in a homologous recombination pathway gene, such as *BRCA1* or *BRCA2*, is associated with a significantly increased risk of breast, prostate, epithelial ovarian, and exocrine pancreatic cancer. Approximately 20% of ovarian cancer patients, 10% of breast cancer patients, 5% of pancreatic cancer patients, and 0.5%–2% of prostate cancer patients have an underlying *BRCA1* or *BRCA2* mutation.^2-6^ Identification of probands with a pathogenic germline mutation allows for cascade testing of relatives and prevention of future disease through surveillance and/or prophylactic surgery. Furthermore, the identification of a homologous recombination deficiency (HRD)-associated mutation may alter therapeutic options for patients, as Poly-ADP ribose polymerase (PARP) inhibitors are now approved in each of these solid tumor patient populations.^7-11^ For these reasons, universal germline testing of patients with epithelial ovarian cancer and criteria-based testing of certain patients with breast, pancreatic, and prostate cancer, is now recommended.^12^

Tumor genomic profiling via next-generation sequencing (NGS) is becoming an increasingly common tool to guide targeted therapy selection in solid tumor malignancies, and HRD mutations are frequently identified on NGS tests. In one study of 52 426 NGS tests, 20% of ovarian cancers, 15.6% of breast cancers, and 15.4% of pancreatic cancers harbored an HRD mutation.^13^ However, it is thought to be not technically possible to distinguish the somatic versus germline origin of mutations based on tumor-only sequencing. Paired tumor-normal sequencing is the gold standard to differentiate between somatic and germline results, but has significant barriers to real-time implementation.^14,15^ The Association for Molecular Pathology (AMP) and other organizations recently published guidelines suggesting that a variant allele frequency (VAF) of approximately 50% could be considered suspicious for being a germline source in some situations.^16^ However, a study analyzing 1310 cancer patients with paired tumor and germline sequencing found that the VAF of germline mutations fell outside the range suggested by the AMP guidelines in 19% of cases.^17^ To date, there is not a clinically validated mechanism for identifying the origin of pathogenic HRD alterations in tumor-only NGS results. Since distinguishing between somatic and germline origin of these mutations can have important consequences for both treatment options of the patients, as well as for cascade testing and cancer prevention in relatives, there is a need to explore concordance between independently run tumor-only and germline sequencing tests to understand if one type of testing can inform the other.

We sought to describe the correlation of germline and somatic alterations in patients with pancreatic, breast, prostate, and ovarian cancer who received both germline and tumor testing for HRD mutations at a tertiary care academic institution. We also describe the predictive value of a computational method to predict somatic versus germline origin without a patient-matched normal control. This method, called somatic-germline-zygosity (SGZ), measures the mutant allele frequency of variants via deep sequencing and creates a model of genome-wide copy number to characterize alterations.^18^ Since this method does not require a matched normal or fresh tissue, it could be widely used in clinical settings.

## Methods

### IRB Approval and Subject Identification

Approval for this retrospective review of patient records was granted from the Duke University Medical Center (DUMC) Institutional Review Board (IRB) (Pro00101425). The Duke University molecular tumor board, which consists of oncology providers and pathologists, reviews all tumor comprehensive genomic profiling (CGP) results generated within our institution weekly. All CGP results ordered at Duke Cancer Institute are stored both in the electronic medical record as well as an internally developed data warehouse solution called the Molecular Registry of Tumors (MRT).^19^ We used this database to identify all patients with breast, ovarian, pancreatic, and prostate cancer whose tumors were submitted to Foundation Medicine for CGP from 17 March 2014 to 01 February 2020 and who had also undergone germline sequencing. Germline results were obtained through a separate Progeny Clinical database (Progeny Genetics, Aliso Viejo, CA). Patients with breast, ovarian, pancreatic, or prostate cancer who had alterations in *BRCA1*, *BRCA2*, *CHEK2*, *ATM*, *BRIP1*, *RAD51C*, *RAD51D*, *PALB2*, or *CDH1* were identified. Genes were selected for inclusion in this study based on their role in homologous recombination DNA repair, association with hereditary cancer risk for cancers included in this study, and inclusion on somatic and germline NGS panels used at our institution. All alteration types, including substitutions, insertions, deletions, copy number changes, and structural rearrangements, and all reported pathogenicity classifications, including pathogenic, likely pathogenic, and variants of uncertain significance (VUS) were included in the study.

### Germline and Tumor CGP Sequencing Analysis

All germline and tumor CGP sequencing analyses were ordered at the discretion of oncology providers as a component of routine cancer care. All tumor-only CGP analyses were performed by Foundation Medicine (Cambridge, MA), and included results from FoundationOne®, FoundationOne® CDx, and FoundationOne® Liquid panels. Germline sequencing was performed by either Invitae Corporation (San Francisco, CA), Myriad Genetics (Salt Lake City, UT), or Ambry Genetics (Aliso Viejo, CA).

### SGZ Analysis

The somatic germline zygosity (SGZ) algorithm is a computational method for predicting somatic versus germline origin and zygosity (homozygous versus heterozygous) for base substitutions and short indels identified from deep massively parallel sequencing of tumor FFPE specimens without the need for a patient matched normal control. The FoundationOne CDx assay, which captures 324 cancer-related genes and >3500 single nucleotide polymorphisms (SNPs) located throughout the genome, utilizes a comparative genomic hybridization (CGH)-like method to build a patient-specific genome-wide copy number profile. Log-ratios of sequence coverage compared against a process matched heterozygous normal control are normalized and concurrently segmented and interpreted alongside allele frequencies of common SNPs. These inputs are used to estimate ploidy, tumor purity (accounting for incidental admixed normal DNA), and copy number at each segment. Using the model’s predicted ploidy and tumor purity, as well as variant allele frequency (VAF) for a given alteration, SGZ evaluates a germline and a somatic hypothesis using a two-tailed binomial test.^18^

### Data Analysis

Chart review was performed using the electronic medical record to obtain baseline demographic data for patients including race, age, and tumor type. Results of tumor CGP and germline testing, including genes interrogated, date of testing, and sample used, were recorded and maintained using REDCap software (Research Electric Data Capture; Vanderbilt, TN).^20^ Patients who had both germline and tumor testing performed for the genes of interest were included in analysis. The incidence of somatic and germline alterations for these patients was assessed, and concordance between the two results was analyzed using descriptive statistics.

## Results

### Demographic Characteristics

**Figure 1.**
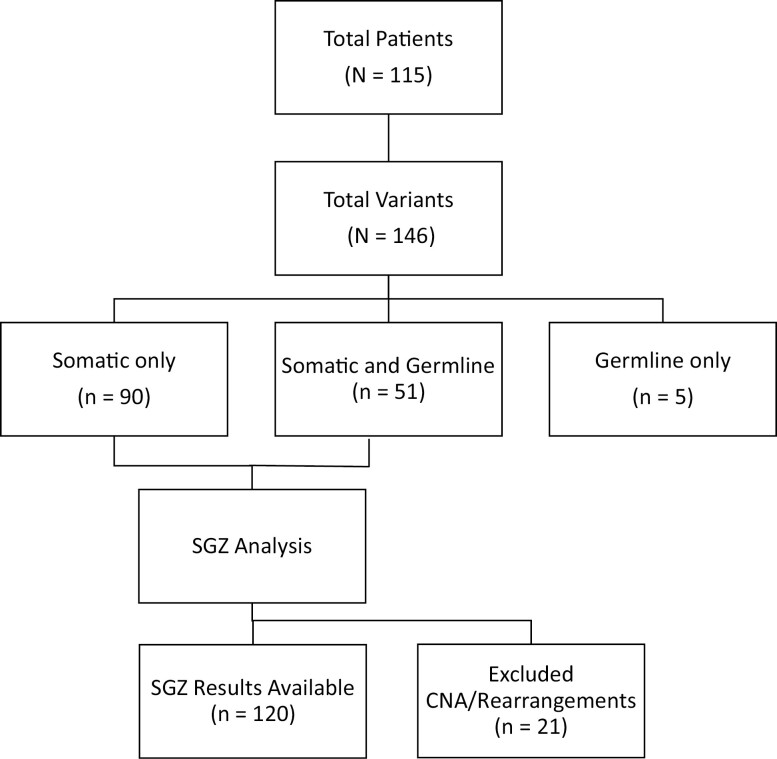
Overview of patient cohort and concordance between germline and tumor sequencing results. A total of 115 patients harboring 146 total alterations were identified and included in the study. The number of alterations identified on tumor-only somatic testing, germline testing, or both somatic and germline testing are shown. A subset of alterations identified on tumor-only somatic testing were also analyzed with the SGZ computational modeling.

A total of 115 patients with both tumor-only CGP and germline genetic testing were identified (Fig. 1). Clinical and demographic characteristics of the cohort are presented in Table 1. A significant majority of the cohort was female (76.5%, *N* = 88) and the majority of patients were white (75.7%, *N* = 87). The median age at diagnosis was 56 (range 24–80) years. The most common cancer type in our cohort was breast (49.6%, *N* = 57), followed by prostate (20.0%, *N* = 20), ovarian (18.3%, *N* = 21), and pancreas (12.2%, *N* = 14).

**Table 1.** Patient demographics (*N* = 115).

Characteristic	No. of patients (%)	Median (range)
Age at diagnosis, years		56 (24-80)
Age at tumor NGS testing, years		62 (26-90)
Gender		
** **Female	88	
** **Male	27	
Race		
** **White	87 (75.7)	
** **Black	23 (20.0)	
** **Asian	2 (1.7)	
** **Not reported	2 (1.7)	
** **American Indian	1 (0.9)	
Primary cancer site		
** **Breast	57 (49.6)	
** **Prostate	23 (20.0)	
** **Ovarian, peritoneal, & fallopian tube	21 (18.3)	
** **Pancreas	14 (12.2)	
Number of somatic mutations of interest		
** **1	93 (80.9)	
** **2	15 (13.9)	
** **3+	7 (5.2)	

Abbreviation: NGS, next-generation sequencing.

### Somatic and Germline Genetic Mutations

**Figure 2.**
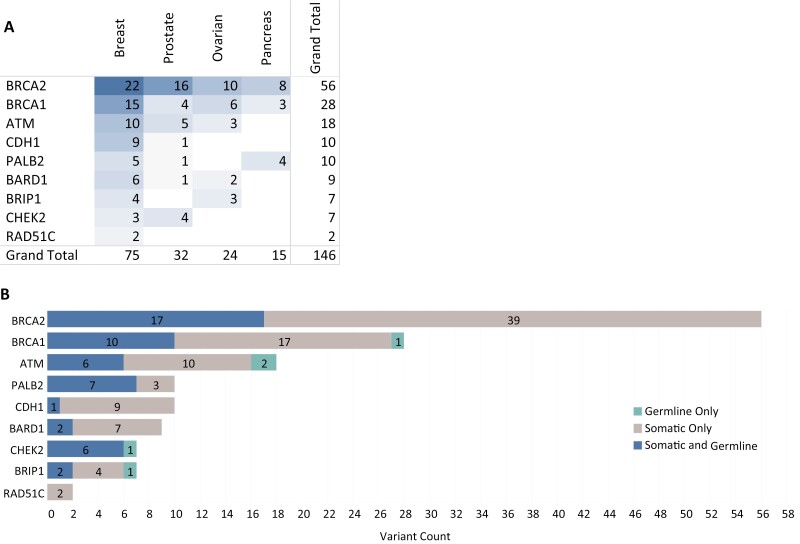
Landscape of alterations detected in genes associated with homologous recombination deficiency and hereditary cancer predisposition (**A**) Genes of interest are listed on the left with the number of alterations detected in the cancer type indicated on top. (**B**) The number of alterations detected on either somatic testing only (gray), both somatic and germline testing (blue) or germline testing only (green) are shown for each gene.

There were 146 mutations identified in genes of interest included on both the tumor and germline testing panels (Fig. 2A). The highest number of variants were reported in *BRCA2* (*N* = 56), *BRCA1* (*N* = 28), and *ATM* (*N* = 18), while the lowest number of variants were reported in *BRIP1* (*N* = 7), *CHEK2* (*N* = 7), and *RAD51C* (*N* = 2). The majority of alterations (61.6%, *N* = 90) were present on tumor testing only, while 34.9% (*N* = 51) of alterations were identified on both tumor and germline testing and 3.4% (*N* = 5) of alterations were identified on germline testing only (Fig. 2B). The proportion of variants that were present on both tumor and germline testing varied widely by gene, with the highest proportion found in *CHEK2* variants (85.7%, *N* = 6) and the lowest proportion found in *RAD51C* variants (0%, *N* = 0). Similarly, the proportion of variants that were present on tumor testing only varied widely by gene, with the highest proportion found in *RAD51C* (100%, *N* = 2) and *CDH1* (90%, *N* = 9) and the lowest proportion found in *CHEK2* (0%, *N* = 0).

In addition, five patients had an alteration in a gene of interest identified on germline testing that was not identified on tumor testing. Three of these patients had prostate cancer while one each had ovarian and breast cancer. Four of these patients harbored VUS alterations in *ATM* (*N* = 2), *BRIP1* (*N* = 1), or *BRCA1* (*N* = 1), while a single patient harbored a low penetrance pathogenic *CHEK2* I157T variant according to the germline testing report. A review of the tumor CGP results revealed that two of these VUS alterations, one in ATM and one in BRIP1, were identified, but classified as benign and filtered from the final report. The other ATM VUS occurred in an intron, outside of canonical splice sites. This alteration was also detected by the tumor CGP test but was filtered from the final report. In addition, one patient was found to harbor a germline duplication of exons 2-3 in *BRCA1* of uncertain significance. Due to differences in assay design, the tumor NGS test would not have been expected to detect this alteration, which is clearly stated in the test technical specifications. Finally, the low penetrance CHEK2 I157T alteration identified via germline testing in a prostate cancer patient was absent from the tumor CGP test, which had sufficient coverage in this region of the CHEK2 gene. The second variant of uncertain significance in POLE detected on the same germline test for this patient was also missing from the tumor CGP results. The reason for this discrepancy is unknown but could suggest a sampling issue or contamination from an alternative source such as clonal hematopoiesis.

### SGZ Analysis

**Figure 3.**
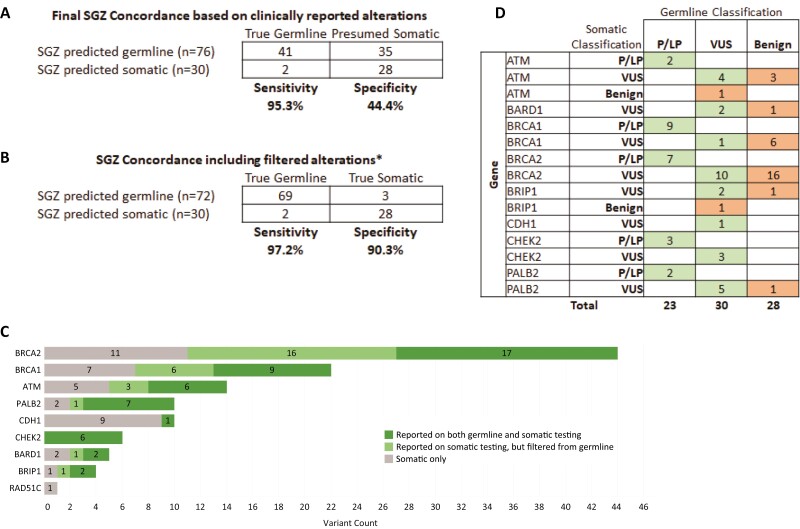
Predictive value of the SGZ algorithm. The sensitivity and specificity of the SGZ algorithm were calculated using either clinically reported alterations (**A**) or additional data including filtered benign variants (**B**). (**C**) The number of alterations in each gene that were reported concordantly on both somatic and germline tests are shown in dark green, the number of alterations that were reported as VUS on somatic testing, but were classified as benign and filtered from germline results are shown in light green, and the number of alterations that were reported on somatic test only are shown in gray. (**D**) Comparison of variant classification for alterations detected on both germline and somatic testing. Concordant classifications are shaded green while discordant classification are shaded orange. *4 variants were excluded due to lack of access to filtered variants.

We next determined the ability of the SGZ algorithm to accurately predict the origin of alterations detected on tumor-only CGP sequencing. As the SGZ algorithm was only designed to predict the origin of single nucleotide or short insertion/deletion variants, 21 copy number alterations and structural rearrangements were excluded from this analysis, leaving 120 total alterations. For this analysis, germline testing was considered the gold standard, with any alteration detected on germline testing considered true germline, while any alteration not reported was presumed somatic. The SGZ algorithm was able to generate a prediction for 88.3% (106/120) of alterations. We then compared the SGZ prediction to germline testing results based on clinically reported variants and found the algorithm had a high sensitivity of 95.3% to predict germline alterations, with only two true germline alterations predicted to be somatic (Fig. 3A).

However, a preliminary calculation of the specificity of the algorithm was only 44.5%, predominately driven by alterations predicted to be germline based on the SGZ algorithm, but that were not reported clinically on the germline testing report. A closer examination of these alterations revealed that many were classified as VUS on the tumor-only testing report, but were classified as benign in ClinVar (data not shown). One source of discordance between the SGZ prediction and germline testing results may be the differences in variant classification and filtering, with the germline testing companies filtering out variants they classified as benign from the final clinical report. To determine if this was the case, we obtained additional data from the germline sequencing company for 41 of the 45 presumed somatic alterations classified as VUS on the tumor-only sequencing reports. Of these 41 presumed somatic mutations, we found that 28 had been identified on germline sequencing, but classified as benign and filtered from the final clinical report (Fig. 3C). Overall, for 81 variants with both a germline and somatic variant classification available, 37.0% (*n* = 30) were found to be discrepant (Fig. 3D). Limiting to only pathogenic alterations, there was 100% concordance (*n* = 23) between somatic and germline classifications. After incorporating this new data, we found that the specificity of the SGZ algorithm greatly improved from 44.4% to 90.3% (Fig. 3B), with only three predicted germline alterations actually being true somatic alterations and two predicted somatic alterations being true germline alterations. This correlates to a positive predictive value of 95.8% for germline alterations and 93.3% for somatic alterations.

**Figure 4.**
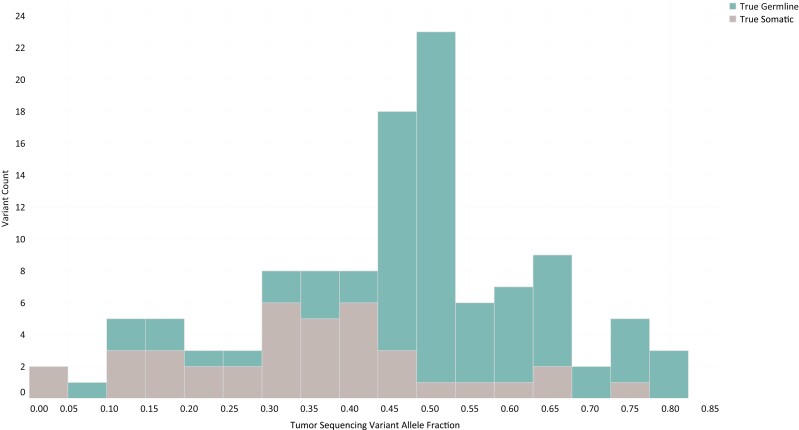
Tumor sequencing variant allele fractions. Histogram displaying variant allele fractions derived from tumor sequencing for known germline (green) or somatic (gray) variants.

Finally, we examined tumor sequencing variant allele frequencies (VAFs) for the 116 variants included in the SZG analysis above. While the average VAF for somatic variants was significantly lower than that for germline variants (0.35 vs. 0.50, *P* = .000008), 15.4% (12/78) of germline alterations had a VAF outside the predicted range for a germline mutation (Fig. 4).^16,17^

## Discussion

Comprehensive genomic profiling of tumors via NGS is becoming an increasingly common tool to guide targeted therapy selection, especially for patients with advanced or metastatic disease. In the absence of paired sequencing of matched normal tissue, there is no clinically validated method to accurately determine if an alteration detected via tumor sequencing originated in the germline or was acquired somatically during tumor evolution. While paired tumor-normal sequencing would be ideal, it is not feasible in many clinical settings due to resource and process limitations. Thus, in the real world, cancer patients more commonly receive independently run germline and tumor sequencing analysis; our study was designed to understand how results from these separate tests compared to each other. We were able to determine that 36.1% (51/141) of alterations in *BRCA1*, *BRCA2*, *ATM*, *CDH1*, *PALB2*, *BARD1*, *BRIP1*, *CHEK2*, and *RAD51C* were identified on tumor sequencing tests in patients with breast, prostate, ovarian, and pancreas cancer were also identified on germline tests indicating that tumor-only CGP does have some utility in discovering clinically relevant inherited alterations in addition to somatic drivers. We also found that the SGZ computational algorithm had a 97.2% sensitivity and 90.3% specificity for accurately predicting somatic versus germline status. Given this high sensitivity for accurately predicting somatic versus germline status without the need for a matched normal control, the SGZ prediction has a potential for informing clinical decision making and prioritizing patients for follow-up germline testing. Specifically, identification of a predicted germline alteration on a tumor CGP test could be useful in prioritizing patients for follow-up germline testing, especially in cases where the patient would not otherwise meet guideline recommended criteria for a genetics referral. This is supported by results from a separate study that found that 9.7% (101/1040) of patients who harbored pathogenic germline mutations identified through paired sequencing of tumor and germline DNA would not have met the criteria for germline testing based on clinical guidelines.^21^ Conversely, in some situations providers might incorrectly assume a pathogenic HRD mutation identified on tumor testing is germline without further confirmation. In cases such as this, a somatic SGZ prediction might be helpful in guiding a provider toward ordering a confirmatory germline sequencing test.

However, our study also identified several limitations to using tumor testing alone to inform the need for subsequent germline testing. First, we identified a small number of cases where the SGZ algorithm incorrectly predicted alteration origin (4.9%, 5/102). In consensus with the analytic validation of SGZ,^22^ discordances could largely be explained with only a few error modes. In addition, there are other factors that would impact the algorithm callability such as suboptimal copy number profile generation (typically due to over segmentation) and extremes in tumor content. When tumor content is sufficiently high, the signal from the normal DNA is dampened or when tumor content is sufficiently low, the signal from the tumor is dampened. Furthermore, since the accuracy of the SZG algorithm is mostly dependent on parameters related to tumor content, sequencing coverage depth, and optimal copy number profile generation, we predict this algorithm could be used for other genes associated with hereditary cancer risk that were not included in this study, although this would require further validation.

We also found that a significant source of discrepancy between germline and tumor sequencing assays was differences in variant interpretation and filtering practices between laboratories. The reasons for this are complex. Germline testing companies generally classify variants in accordance with guidelines proposed by the American College of Medical Genetics and Genomics (ACMG),^22^ but there are no widely adopted guidelines for the classification of somatic sequence variants.^23^ Conversely, tumor testing platforms generally follow reporting guidelines to benefit patients with an active cancer diagnosis. Of note, concordance between the classification of pathogenic and likely pathogenic alterations was 100% and discrepancies were limited to variants classified as VUS or benign in our study, which are widely recommended to not be considered to inform clinical decision-making.

Finally, we found a small number of cases (4.3%, 5/115) where HRD alterations were detected on germline testing alone. A review of these cases found that technical issues including differences in variant classification and filtering as well as assay design contributed to these discrepancies.

Taken together, these observations suggest that tumor and germline sequencing tests should be considered complementary and that a negative result on one test type does not obviate the need for the other test type. In our study, we found that the SGZ algorithm had high sensitivity, specificity, and predictive power to identify potential germline mutations in HRD genes from tumor only testing, so could be useful is identifying patients for follow-up germline testing, especially in patients who would not have otherwise been referred. However, we also identified a significant number of cases where germline mutations were missed by tumor CGP tests or were incorrectly predicted to be somatic variants. In cases like this, patients should still be referred for germline testing if they otherwise meet guideline recommended criteria. Finally, providers should be aware that there are multiple potential reasons for discordance between tumor and germline testing results, and in cases where discrepancies are noted, should contact the testing laboratories for clarification or seek guidance from medical geneticists, genetic counselors, or molecular tumor boards.

## Data Availability

The data underlying this article cannot be shared publicly due to patient privacy concerns. The data will be shared on reasonable request to the corresponding author.
